# Probe-Based Confocal Laser Endomicroscopy During Immune Checkpoint Inhibitor Therapy: A Translational Framework Linking Intestinal Barrier Function, Microbiota, and Clinical Outcomes

**DOI:** 10.3390/nu18183086

**Published:** 2026-09-20

**Authors:** Paola Spessotto, Francesca Orbosuè, Maurizio Mongiat, Stefania Maiero, Marco Sartori, Michela Guardascione, Luisa Foltran, Arianna Fumagalli, Marco de Scordilli, Alessandra Bearz, Lucia Fratino, Silvia Bolzonello, Michele Spina, Fabio Puglisi, Vincenzo Canzonieri, Renato Cannizzaro, Riccardo Dolcetti, Elena Ongaro, Stefano Realdon

**Affiliations:** 1 Molecular Oncology Unit, Centro di Riferimento Oncologico Aviano, (CRO) IRCCS, 33081 Aviano, Italy; mmongiat@cro.it; 2 Oncological Gastroenterology Unit, Department of Medical Oncology, Centro di Riferimento Oncologico Aviano, (CRO) IRCCS, 33081 Aviano, Italy; francesca.orbosue@cro.it (F.O.); smaiero@cro.it (S.M.); marco.sartori@cro.it (M.S.); rcannizzaro@cro.it (R.C.); stefano.realdon@cro.it (S.R.); 3 Medical Oncology and Cancer Prevention, Department of Medical Oncology, Centro di Riferimento Oncologico Aviano, (CRO) IRCCS, 33081 Aviano, Italy; michela.guardascione@cro.it (M.G.); luisa.foltran@cro.it (L.F.); arianna.fumagalli@cro.it (A.F.); marco.descordilli@cro.it (M.d.S.); silvia.bolzonello@cro.it (S.B.); fabio.puglisi@cro.it (F.P.); elena.ongaro@cro.it (E.O.); 4 Medical Oncology and Immune-Related Tumors, Department of Medical Oncology, Centro di Riferimento Oncologico Aviano, (CRO) IRCCS, 33081 Aviano, Italy; alessandra.bearz@cro.it (A.B.); lfratino@cro.it (L.F.); mspina@cro.it (M.S.); 5 Department of Medicine, University of Udine, 33100 Udine, Italy; 6 Pathology Unit, Centro di Riferimento Oncologico Aviano, (CRO) IRCCS, 33081 Aviano, Italy; 7 Department of Medical, Surgical and Health Sciences, University of Trieste, 34127 Trieste, Italy; 8 Peter MacCallum Cancer Center, Melbourne, VIC 3000, Australia; riccardo.dolcetti@petermac.org; 9 Sir Peter MacCallum Department of Oncology, The University of Melbourne, Melbourne, VIC 3010, Australia; 10 Department of Microbiology and Immunology, The University of Melbourne, Melbourne, VIC 3010, Australia

**Keywords:** gut microbiota, immune checkpoint inhibitors, pCLE, gut permeability, gastrointestinal toxicity, immune-related adverse events

## Abstract

Immune checkpoint inhibitors (ICIs) have transformed the treatment of several malignancies, but their efficacy and gastrointestinal (GI) immune-related adverse events (irAEs) vary considerably among patients. Growing evidence suggests that the gut microbiota and intestinal barrier integrity are associated with both therapeutic response and toxicity, although their causal contribution and clinical utility remain incompletely defined. Dysbiosis may disrupt epithelial homeostasis, increase intestinal permeability, and promote mucosal immune activation, potentially influencing antitumor immunity and susceptibility to ICI-induced colitis. Current biomarkers of intestinal permeability are limited by their indirect nature, lack of standardization, or poor spatial resolution. Probe-based confocal laser endomicroscopy (pCLE) enables real-time, in vivo visualization of epithelial abnormalities associated with barrier dysfunction, including fluorescein leakage, epithelial gaps, and cell shedding. In parallel, dietary and microbiota-directed interventions are being investigated as potential strategies to modulate intestinal homeostasis during ICI therapy, although evidence for their clinical benefit and effects on intestinal barrier integrity remains limited. This review summarizes current evidence linking the gut microbiota, intestinal barrier dysfunction, and cancer immunotherapy and discusses the potential role of pCLE as a candidate functional imaging tool in ICI-treated patients. We highlight current knowledge gaps and propose a translational framework integrating pCLE with microbiome profiling, circulating biomarkers, histopathology, and clinical outcomes to determine whether this approach can improve patient stratification and the early identification and monitoring of GI toxicity.

## 1. Introduction

The advent of immune checkpoint inhibitors (ICIs) targeting programmed cell death protein 1 (PD-1), programmed death-ligand 1 (PD-L1), and cytotoxic T-lymphocyte-associated protein 4 (CTLA-4) has revolutionized the treatment of a broad spectrum of malignancies [1,2,3]. By releasing inhibitory signals that restrain T-cell activation, these agents restore antitumor immunity and have produced durable clinical responses in subsets of patients with melanoma, lung cancer, renal cell carcinoma, and several other malignancies [4,5]. Despite these remarkable advances, only a proportion of patients derive long-term clinical benefit, as some exhibit primary resistance, whereas others eventually develop acquired resistance following an initial response [4]. In parallel, the nonspecific activation of the immune system can trigger immune-related adverse events (irAEs), among which gastrointestinal (GI) toxicity is one of the most frequent and clinically significant complications [6,7,8,9]. Immune-mediated enterocolitis often requires treatment interruption, systemic corticosteroids or biological therapies, thereby negatively affecting patients’ quality of life and, in some cases, limiting the continuation of anticancer therapy [10].

Understanding the factors that determine both therapeutic efficacy and toxicity therefore represents one of the major unmet needs in cancer immunotherapy. Among the various host-related determinants, the gut microbiota has emerged as a critical regulator of ICI outcomes [11,12,13,14]. Accumulating evidence demonstrates that microbial diversity, specific bacterial taxa, and microbiota-derived metabolites influence antitumor immune responses and are associated with both clinical response and susceptibility to GI irAEs [15,16,17,18,19,20]. These observations indicate that the intestinal ecosystem is not merely a passive bystander but an active modulator of systemic immunity. Because the intestinal microbiota is highly responsive to environmental factors, particularly diet, nutritional interventions have emerged as attractive, non-invasive strategies to modulate microbial composition, preserve epithelial barrier integrity, and potentially improve the efficacy and tolerability of cancer immunotherapy [21]. This concept provides the biological rationale for microbiota-targeted approaches, including probiotics, postbiotics, dietary modulation, and fecal microbiota transplantation [22,23,24].

The intestinal epithelial barrier represents the functional interface through which the host and gut microbiota continuously communicate. This single-cell-thick epithelium, sealed by tight junction complexes and protected by the mucus layer, regulates the selective passage of nutrients while preventing uncontrolled translocation of luminal microorganisms and their products [25]. Alterations in barrier integrity increase intestinal permeability, facilitating the passage of microbial antigens and metabolites into the underlying mucosa, where they amplify local and systemic immune activation. Accordingly, disruption of the gut barrier has emerged as a key mechanism linking dysbiosis to chronic inflammation and immune dysregulation [26,27].

Interestingly, the pathophysiological mechanisms underlying ICI-induced enterocolitis share several features with inflammatory bowel disease (IBD), including epithelial injury, impaired tight junction integrity, increased intestinal permeability, and exaggerated mucosal immune responses [28]. Beyond its established role in the development of GI toxicity, emerging evidence suggests that intestinal barrier dysfunction may also reflect the systemic immune activation associated with antitumor efficacy. Thus, intestinal barrier dysfunction may represent one component of a complex and potentially bidirectional relationship among microbiota composition, mucosal immune activation, antitumor response, and immune-related toxicity. Whether barrier dysfunction precedes these clinical outcomes, results from immune activation, or reflects both processes remains to be established in longitudinal studies.

Although several biomarkers have been proposed to assess intestinal permeability, including circulating proteins, fecal biomarkers, and sugar absorption tests, most provide indirect measurements, are technically demanding, or lack sufficient sensitivity and spatial resolution for routine clinical application. Consequently, there remains a critical need for clinically applicable methods capable of directly evaluating epithelial barrier function in vivo. Importantly, although the gut microbiota has emerged as a major determinant of response and toxicity in patients receiving ICIs, the contribution of intestinal barrier integrity and permeability has received comparatively little attention [29]. Consequently, technologies capable of providing a direct, functional in vivo assessment of epithelial barrier integrity remain largely unexplored in cancer immunotherapy.

Probe-based confocal laser endomicroscopy (pCLE) offers a unique opportunity to address this unmet need. By combining intravenous fluorescein administration with high-resolution laser endomicroscopy during standard endoscopic procedures, pCLE enables real-time visualization of tissue architecture at the cellular level [30,31,32,33]. The technique allows real-time visualization of epithelial cell shedding, fluorescein leakage, crypt architecture, vascular alterations, and epithelial gaps, all features that have been associated with increased permeability in inflammatory disorders [34,35,36]. These characteristics make pCLE a promising tool for investigating intestinal barrier dysfunction in cancer immunotherapy.

In this mini-review, we summarize current evidence linking gut microbiota, intestinal permeability, and cancer immunotherapy, with particular emphasis on the emerging role of pCLE as a functional imaging tool for evaluating epithelial barrier integrity. Rather than reviewing an established clinical application, this article proposes a translational framework in which pCLE could be investigated as a candidate functional imaging biomarker of intestinal barrier integrity, complementing microbiome profiling and other molecular biomarkers to improve the prediction of therapeutic efficacy and GI toxicity during ICI treatment. However, direct evidence linking gut microbiota alterations, pCLE-detected barrier abnormalities, and clinical outcomes during ICI therapy is currently lacking. Accordingly, this integrated approach should be considered a hypothesis-generating framework for future prospective investigation rather than an established biological or clinical pathway.

## 2. The Gut Barrier During Immune Checkpoint Inhibitor Therapy

The intestinal barrier constitutes the dynamic interface between the host immune system and the luminal environment, which harbors trillions of microorganisms together with dietary antigens, metabolites, and potential pathogens. Its primary function is to maintain a selective permeability that permits nutrient absorption while preventing the uncontrolled translocation of microorganisms and harmful luminal components. Structurally, the barrier is composed of a physical and a chemical component [25]. The physical barrier consists of a single layer of intestinal epithelial cells interconnected by tight junctions, adherens junctions, and desmosomes, which tightly regulate paracellular permeability. Among these, tight junctions represent the principal determinants of epithelial integrity and barrier function [37]. The chemical barrier is formed by the mucus layer, antimicrobial peptides, and secretory IgA, which collectively limit microbial adhesion and invasion while preserving immune homeostasis [38].

Beyond these structural components, the gut microbiota plays a pivotal role in maintaining barrier integrity. Microbiota-derived metabolites, particularly short-chain fatty acids (SCFAs) such as butyrate, acetate, and propionate, enhance epithelial barrier function by promoting tight junction assembly, epithelial repair, and mucosal immune tolerance [22]. Other microbial metabolites, including indole derivatives, conjugated fatty acids, and bile acids, further modulate epithelial permeability through interactions with epithelial and immune cells [39,40,41,42]. Conversely, dysbiosis alters the production of these bioactive metabolites, impairs epithelial integrity, and increases intestinal permeability, thereby facilitating microbial translocation and chronic immune activation. Consequently, intestinal barrier dysfunction represents a critical mechanistic link between alterations in the gut microbiota and the development of inflammatory and immune-mediated diseases.

In patients receiving ICIs, disruption of the finely regulated host–microbiota dialogue may compromise epithelial barrier integrity, thereby contributing not only to immune-related GI toxicity but also to the modulation of systemic antitumor immunity. Among GI irAEs, immune checkpoint inhibitor-associated colitis (ICI colitis) is one of the most frequent and clinically relevant complications, occurring particularly in patients treated with combined anti-CTLA-4 and anti-PD-1 regimens [6,9]. Beyond its clinical impact, ICI colitis represents a valuable model for investigating the interplay between immune activation, the gut microbiota, and epithelial barrier dysfunction.

Although the pathogenesis of ICI colitis has not yet been fully elucidated, accumulating evidence indicates that excessive immune activation, microbial dysbiosis, and epithelial barrier disruption act synergistically to promote intestinal inflammation. Blockade of PD-1 and CTLA-4 signaling enhances T-cell activation and restores antitumor immunity but may also impair peripheral immune tolerance within the intestinal mucosa. Single-cell transcriptomic analyses have revealed profound alterations in tissue-resident CD8^+^ and CD4^+^ T cells, accompanied by activation of myeloid, stromal, and epithelial cell populations, suggesting that multiple cellular compartments contribute to disease development [43,44,45,46]. These immune alterations are associated with increased production of pro-inflammatory cytokines, epithelial injury, impaired tight junction integrity, and enhanced intestinal permeability, thereby facilitating microbial translocation and amplifying local inflammatory responses [47,48,49,50].

Despite these advances, the precise molecular mechanisms linking immune activation to epithelial barrier dysfunction remain incompletely understood. In particular, whether barrier disruption represents merely a consequence of intestinal inflammation or an early event predisposing to ICI-induced colitis has yet to be determined. This distinction has important clinical implications because early identification of barrier dysfunction could facilitate patient stratification and enable preventive interventions before the onset of clinically overt toxicity.

Current therapeutic strategies for ICI colitis, including corticosteroids, anti-TNF agents, vedolizumab, and, more recently, fecal microbiota transplantation, have largely been adapted from IBS management [51,52,53,54,55,56,57]. However, important biological differences exist between these two conditions, and a substantial proportion of patients develop steroid-refractory disease or experience recurrent symptoms. These limitations underscore the need for a better understanding of intestinal barrier biology during cancer immunotherapy and for the development of biomarkers capable of identifying patients at risk of GI toxicity before irreversible mucosal damage occurs.

Collectively, these observations support the concept that intestinal barrier dysfunction is not only a pathological consequence of ICI-induced inflammation but may also represent an early and measurable event preceding overt colitis. However, current clinical assessment relies primarily on symptoms, endoscopy, histology, and fecal inflammatory markers, which provide limited information on epithelial barrier function itself. This gap highlights the need for functional imaging approaches capable of directly evaluating intestinal permeability in vivo. The direct functional assessment provided by pCLE may represent a valuable biomarker during ICI therapy.

## 3. Gut Microbiota as a Regulator of ICI Response and Toxicity

Several studies have reported associations between the gut microbiome and clinical outcomes during ICI therapy, particularly in patients diagnosed with melanoma, non-small cell lung carcinoma (NSCLC), and hepatocellular carcinoma (HCC) [20,52,58,59,60,61,62,63]. Therefore, modulation of the gut microbiome has emerged as a promising strategy to enhance immunotherapy efficacy, also due to the encouraging results in preclinical models [64,65]. Greater microbial diversity and enrichment of selected bacterial taxa have been associated with response in some cohorts, whereas distinct microbiota profiles have also been linked to GI toxicity. However, the taxa identified across studies are not fully consistent, and differences in geography, diet, antibiotic exposure, tumor type, treatment regimen, sequencing methods, and bioinformatic pipelines limit reproducibility and clinical translation. These findings therefore support the gut microbiome as a potentially relevant modulator and biomarker of ICI outcomes, but they do not yet define a universally favorable microbial signature. Interventional approaches, including dietary modulation, live biotherapeutics, and fecal microbiota transplantation, are under investigation, with encouraging but still preliminary clinical results. Importantly, microbiome composition alone may provide an incomplete representation of host–microbe interactions. Similar microbial profiles may have different functional consequences depending on microbial metabolism, mucus integrity, epithelial permeability, and the underlying mucosal immune state. Integrating compositional microbiome data with a functional assessment of the intestinal barrier may therefore provide a more biologically informative approach than either measurement alone.

### Diet as a Modulator of the Gut Microbiota-Barrier Axis During Immunotherapy

Diet is one of the most powerful and readily modifiable factors influencing the composition and metabolic activity of the gut microbiota, thereby shaping intestinal barrier integrity and mucosal immune homeostasis. Dietary patterns rich in plant-based foods, dietary fiber, and polyphenols promote microbial diversity and enrich beneficial bacterial taxa capable of producing SCFAs, particularly butyrate, acetate, and propionate [12,66]. These metabolites enhance epithelial barrier function by promoting tight junction assembly, stimulating mucus production, supporting epithelial repair, and inducing regulatory immune responses [67,68]. In contrast, Western-style diets characterized by high consumption of saturated fats, animal proteins, refined sugars, and low fiber intake are associated with gut microbiota dysbiosis, impaired epithelial barrier function, increased intestinal permeability, and chronic low-grade inflammation, all of which may contribute to intestinal immune dysregulation and increase the risk of inflammatory disorders, including IBS [69].

Beyond shaping the microbiota, specific nutritional interventions may directly support epithelial barrier function. Glutamine, the primary metabolic fuel for enterocytes, is essential for maintaining epithelial integrity, and oral supplementation has been shown to improve intestinal permeability in patients with post-infectious diarrhea-predominant IBS, highlighting the potential of barrier-targeted nutritional strategies [70]. Although these findings cannot be directly extrapolated to patients receiving ICIs, they provide proof of principle that selected nutritional interventions may influence epithelial barrier function. Interestingly, emerging clinical studies suggest that dietary habits may influence both the efficacy and tolerability of immunotherapy. In particular, higher dietary fiber intake has been associated with improved responses to anti-PD-1 therapy, whereas unnecessary antibiotic exposure and other factors that disrupt the gut microbiota may adversely affect treatment outcomes [71,72].

Collectively, these findings provide a biological rationale for investigating dietary modulation as a non-pharmacological strategy to support microbial diversity and intestinal barrier homeostasis during ICI therapy. Importantly, no clinical evidence currently demonstrates that dietary or microbiota-directed interventions modify pCLE-defined barrier abnormalities in patients receiving ICIs, or that any clinical benefit occurs through restoration of pCLE-detected barrier integrity. Prospective interventional studies are therefore required before specific dietary, probiotic, or postbiotic strategies can be recommended as part of routine oncological care. Whether dietary interventions effectively restore epithelial barrier integrity could ultimately be monitored using functional imaging approaches such as pCLE.

## 4. Current Approaches to the Assessment of Intestinal Barrier Function

Given the central role of intestinal barrier dysfunction in regulating host–microbiota interactions, mucosal immune homeostasis, and the development of immune-related GI toxicity, reliable assessment of epithelial barrier integrity has become an area of growing clinical interest. An ideal biomarker should provide a direct, reproducible, and minimally invasive evaluation of intestinal permeability, allowing early identification of epithelial dysfunction before the onset of overt inflammation. Although several approaches have been developed, each assesses different aspects of barrier function and presents important methodological limitations that currently restrict their routine clinical application.

Among non-invasive methods, the lactulose-to-mannitol (L) urinary excretion test remains the most widely used functional assay of intestinal permeability. Under physiological conditions, the intact intestinal epithelium preferentially absorbs the smaller molecule mannitol, whereas lactulose is minimally absorbed, resulting in a low urinary L ratio. Increased paracellular permeability facilitates lactulose absorption, leading to an elevated L ratio. However, differences in sugar formulations, dosing regimens, urine collection protocols, and analytical methodologies have limited the standardization, reproducibility, and comparability of this test across clinical studies [73,74,75].

Blood-based biomarkers have also been proposed as surrogate indicators of epithelial integrity. Intestinal fatty acid-binding protein (I-FABP), a cytosolic protein released following enterocyte injury, reflects acute epithelial damage, whereas plasma citrulline, synthesized by small intestinal enterocytes, serves as a marker of enterocyte mass and absorptive capacity [75,76,77]. Nevertheless, these biomarkers provide only indirect information on barrier function, as they primarily reflect epithelial injury or enterocyte loss rather than permeability itself, and their levels may be influenced by several pathological conditions unrelated to intestinal barrier dysfunction.

Ex vivo approaches, including immunohistochemical analysis of tight junction proteins and adherens junction components, as well as measurement of transepithelial electrical resistance using Ussing chambers, provide valuable mechanistic insights into epithelial integrity [78,79]. However, these techniques require intestinal biopsy specimens, are labor-intensive, and are restricted to specialized research laboratories, precluding their use for longitudinal monitoring.

In vitro assays include the assessment of permeability by measuring the translocation of FITC across a monolayer of colonic epithelial cells cultured on transwell inserts. In this type of assay, FITC is added to the apical side of the monolayer in combination with supernatants derived from patient biopsy samples [80].

Fecal biomarkers such as calprotectin and zonulin have also been investigated as indicators of intestinal inflammation or barrier dysfunction; however, their specificity for epithelial permeability remains controversial, and they provide no information on the location or extent of barrier disruption.

An ideal biomarker of intestinal barrier function should be minimally invasive, reproducible, capable of providing real-time functional information, and able to identify early epithelial alterations before the onset of overt mucosal inflammation. At present, none of the available approaches fully satisfies these requirements.

Overall, currently available methods provide either indirect, static, or ex vivo measurements of intestinal barrier integrity and are unable to capture the dynamic and spatially heterogeneous nature of epithelial permeability in vivo. Their limited standardization, inability to localize barrier defects, and poor suitability for repeated clinical assessment underscore the need for innovative approaches capable of directly visualizing epithelial barrier function in real time. In this context, pCLE has emerged as a promising technology with the potential to overcome many of these limitations.

## 5. Probe-Based Confocal Laser Endomicroscopy: From Optical Biopsy to Functional Assessment of Intestinal Barrier Integrity

pCLE (probe-based Confocal Laser Endomicroscopy) is an advanced endoscopic imaging technology that enables real-time, in vivo microscopic visualization of the GI mucosa during standard endoscopic procedures. Following intravenous administration of fluorescein sodium, a low-molecular-weight fluorescent contrast agent that diffuses through the vascular and extracellular compartments without entering intact epithelial cells, pCLE generates high-resolution images at nearly histological resolution [81,82,83,84,85,86]. This “optical biopsy” approach permits dynamic evaluation of tissue architecture without the need for tissue removal, providing immediate information on epithelial morphology, vascular organization, and cellular alterations.

Initially developed for the characterization of GI neoplasia, pCLE has progressively evolved from a purely diagnostic imaging modality to a functional tool capable of investigating dynamic biological processes within the tumor microenvironment. One of the earliest and most successful applications has been the evaluation of tumor angiogenesis. By exploiting the intravascular distribution of fluorescein, pCLE allows direct visualization of the microvascular network, including vessel architecture, tortuosity, dilation, leakage, and blood flow. Our group was among the first to demonstrate that these vascular abnormalities can be quantified to generate angiogenic scores reflecting tumor vascularization and permeability in GI cancers [30,31,33,87]. Importantly, pCLE-derived vascular patterns correlated with histopathological angiogenic markers and clinical outcomes [33], supporting the use of real-time endomicroscopy as a functional biomarker of tumor biology rather than solely a morphological imaging technique.

Beyond vascular imaging, pCLE has also demonstrated considerable potential for the real-time assessment of tissue cytoarchitecture. In ovarian cancer, our group showed that pCLE accurately identifies distinctive cellular and architectural features of malignant lesions, enabling intraoperative discrimination between normal and neoplastic tissues [32,88]. These findings highlighted the ability of pCLE to guide surgical decision-making while reducing dependence on conventional frozen-section pathology. Collectively, these studies established pCLE as a versatile translational imaging platform capable of simultaneously evaluating vascular, architectural, and cellular characteristics of human tissues in vivo.

More recently, pCLE has emerged as a valuable tool for the functional assessment of intestinal barrier integrity. Unlike conventional white-light endoscopy, which primarily detects established mucosal inflammation and structural lesions, pCLE enables real-time visualization of subtle epithelial abnormalities that, in other GI settings, have been associated with barrier dysfunction and may precede macroscopic or histological changes. Following intravenous fluorescein administration, disruption of the epithelial barrier is identified by paracellular fluorescein leakage into the intestinal lumen and is frequently accompanied by epithelial cell shedding, epithelial gaps, crypt distortion, and alterations in villous architecture, all of which are recognized hallmarks of increased intestinal permeability [36].

To standardize the evaluation of barrier dysfunction, site-specific scoring systems have been developed. In the terminal ileum, epithelial integrity is commonly assessed using the Watson score, which classifies barrier alterations into three categories: grade I (normal barrier), characterized by physiological shedding of isolated epithelial cells without detectable fluorescein leakage; grade II (functional barrier defect), defined by increased epithelial cell shedding associated with focal fluorescein leakage into the intestinal lumen; and grade III (structural barrier defect), characterized by the presence of microerosions accompanied by extensive fluorescein leakage [34]. In the colon, barrier integrity is primarily evaluated by the morphology of the colonic crypts. Under physiological conditions, intact crypts display a dark central lumen because fluorescein remains confined to the lamina propria, whereas disruption of the epithelial barrier allows fluorescein to enter the crypt lumen, resulting in increased luminal fluorescence and a bright appearance of the crypt opening [34]. Together, these imaging features provide a spatially resolved, real-time assessment of imaging features associated with epithelial barrier dysfunction that cannot be achieved with conventional endoscopy alone. Importantly, these pCLE findings provide functional imaging surrogates of epithelial barrier dysfunction, reflecting altered tight junction integrity, epithelial cell extrusion, and increased paracellular permeability induced by immune activation and host–microbiota interactions. Of note, fluorescein leakage should be regarded as an imaging surrogate of altered barrier integrity rather than a direct quantitative measurement of transepithelial permeability. Its relationship with established functional assays and molecular markers requires further validation, particularly in patients receiving ICIs.

Importantly, in patients with IBD, these microscopic abnormalities correlate with increased intestinal permeability and have been shown to predict clinical relapse, even in individuals who have achieved endoscopic remission [28,89,90,91]. These observations demonstrate that pCLE provides a dynamic functional assessment of epithelial barrier integrity, enabling the detection of subtle mucosal alterations that may precede overt structural damage. This capability makes pCLE particularly attractive for investigating diseases in which epithelial barrier dysfunction is believed to play a pathogenic role. One such setting is cancer immunotherapy. Although increasing evidence indicates that disruption of the intestinal barrier contributes to both immune-related GI toxicity and the modulation of antitumor immune responses during ICI therapy, no studies have yet systematically evaluated epithelial permeability using pCLE in this patient population. By enabling direct, real-time visualization of barrier function in vivo, pCLE represents a promising translational platform for investigating whether early epithelial alterations can be identified before clinically overt ICI-related colitis, integrating functional imaging with microbiome profiling, and developing novel biomarkers to predict both therapeutic efficacy and immune-related GI toxicity in patients receiving ICIs.

### 5.1. pCLE in ICI-Induced Colitis: Proof-of-Concept Observations

To date, the application of pCLE to intestinal barrier assessment in patients receiving immune checkpoint inhibitors (ICIs) has not been systematically investigated. In this context, our ongoing exploratory observational experience provides preliminary examples of the feasibility of integrating pCLE with conventional endoscopic evaluation in patients who develop ICI-associated gastrointestinal (GI) toxicity. These observations are not intended to establish diagnostic or predictive performance, but rather to illustrate the type of complementary information that pCLE may provide when conventional endoscopy and histopathology are performed.

**Figure 1:**
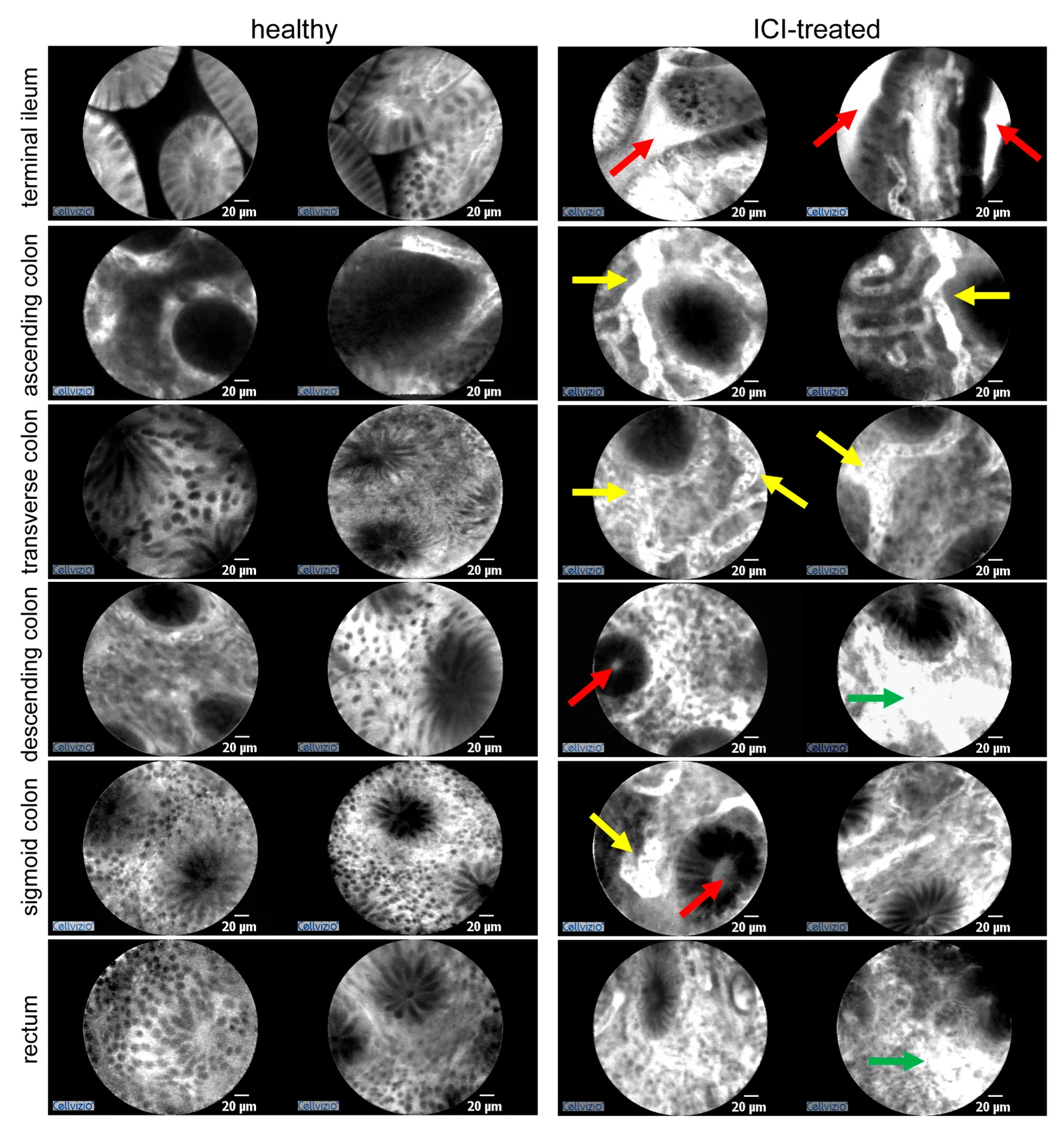
Representative probe-based confocal laser endomicroscopy (pCLE) images of the terminal ileum and different colonic segments in healthy individuals and patients receiving immune checkpoint inhibitors (ICIs). Representative images, obtained from the preliminary proof-of-concept clinical observations, from the terminal ileum, ascending colon, transverse colon, descending colon, sigmoid colon, and rectum illustrate the normal pCLE appearance of the intestinal mucosa compared with the microscopic alterations observed during ICI-induced GI toxicity. Images were acquired using the same intravenous fluorescein administration protocol and comparable timing after fluorescein injection. Healthy mucosa is characterized by preserved villous and crypt architecture, regular microvascular organization, and the absence of fluorescein extravasation. For each colonic segment, two representative images from two healthy individuals are shown (left and right columns, respectively). In contrast, pCLE images from ICI-treated patients demonstrate epithelial barrier permeability (red arrows), characterized by abnormal fluorescein permeation into the villous interstitium in the terminal ileum or into the crypt/glandular lumen in the colon, reflecting epithelial barrier disruption; vascular permeability (green arrows), corresponding to fluorescein leakage from the mucosal microvasculature into the surrounding tissue, consistent with inflammatory vascular leakage; and abnormal microvascular architecture (yellow arrows), including dilated, tortuous, and irregular vessels. For each colonic segment, two representative images from two ICI-treated patients are shown (left and right columns, respectively). Together, these findings demonstrate the ability of pCLE to simultaneously evaluate epithelial barrier integrity, vascular permeability, and microvascular abnormalities in vivo, providing a comprehensive functional assessment of intestinal mucosal homeostasis in patients undergoing ICI therapy.

Patients receiving ICIs who developed GI toxicity requiring treatment interruption underwent colonoscopy combined with pCLE imaging. Real-time assessment focused on epithelial fluorescein leakage, crypt architecture, and microvascular alterations, and findings were considered in relation to endoscopic appearance, histological features, and clinical status before and after corticosteroid treatment. Although the cohort is limited and includes patients with different solid malignancies, including melanoma, renal cell carcinoma, non-small cell lung cancer, and head and neck squamous cell carcinoma, several recurrent imaging patterns have been observed. At the time of clinically apparent GI toxicity, pCLE frequently showed epithelial barrier-associated abnormalities, characterized by increased fluorescein leakage and crypt architectural distortion, together with microvascular alterations (Figure 1).

**Figure 2:**
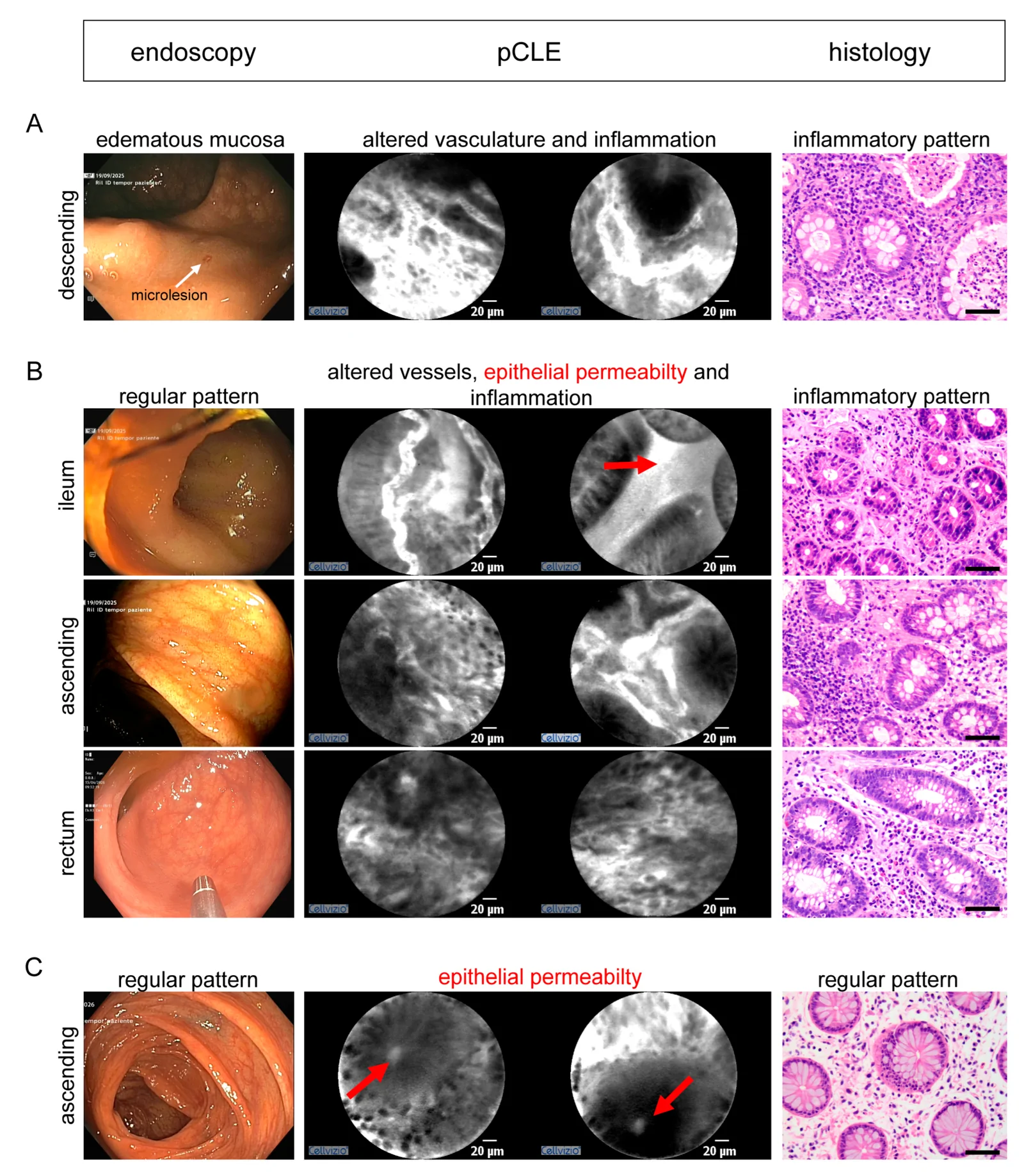
Endoscopic, pCLE, and histological findings in three different patients receiving ICIs. Representative images, obtained from the preliminary proof-of-concept clinical observations, illustrate the complementary diagnostic information provided by pCLE compared with conventional endoscopy and histopathology. (**A**) In a patient with endoscopically evident edematous and ulcerated colonic mucosa, pCLE demonstrates marked inflammatory changes characterized by abnormal microvascular architecture and increased vascular fluorescein leakage, consistent with the corresponding histological findings. (**B**) In a patient with endoscopically unremarkable mucosa, pCLE reveals epithelial barrier permeability together with inflammatory changes, which were confirmed by histological examination. (**C**) In another endoscopically normal colonic segment, pCLE detected epithelial barrier abnormality in the absence of corresponding endoscopic or histological alterations at the time of examination. This finding illustrates the ability of pCLE to identify functional barrier-associated abnormalities that may not be apparent on conventional assessment; however, it does not establish that these alterations precede clinically overt colitis. Red arrows indicate epithelial barrier permeability. Scale bar (histological images), 50 mm.

These abnormalities were also observed in some areas in which conventional endoscopy and histology showed only subtle or equivocal changes (Figure 2).

These observations indicate that pCLE can visualize microscopic fluorescence and architectural features that may not be readily apparent on conventional endoscopy at the time of examination. Importantly, however, the detection of pCLE abnormalities in endoscopically or histologically unremarkable areas should not be interpreted as evidence that these alterations precede clinically overt colitis. Such findings may represent an earlier stage of mucosal injury, a functional alteration occurring independently of overt inflammation, or spatial heterogeneity within the affected mucosa. Longitudinal studies are therefore required to determine their biological and clinical significance.

In selected patients examined during follow-up after corticosteroid treatment, pCLE abnormalities persisted despite apparent endoscopic and histological remission. These observations raise the possibility that barrier-associated alterations may not resolve simultaneously with conventional markers of mucosal inflammation. However, the clinical significance of persistent pCLE abnormalities remains uncertain. In particular, it is currently unknown whether they reflect residual subclinical inflammation, delayed restoration of epithelial integrity, or nonspecific imaging abnormalities, and whether they are associated with subsequent symptom recurrence or other clinical outcomes.

Overall, these preliminary observations support the feasibility of pCLE as a complementary imaging approach for investigating epithelial and microvascular alterations during ICI-associated GI toxicity. They do not, however, establish diagnostic accuracy, predictive value, or a causal relationship between pCLE-detected abnormalities and clinical disease. These questions require prospective longitudinal evaluation.

### 5.2. Potential Clinical Applications: Prediction, Diagnosis, and Monitoring

The potential clinical applications of pCLE can be considered according to the timing of assessment during ICI treatment. Importantly, these applications represent hypotheses arising from the biological rationale and preliminary observations described above and should not be considered established clinical uses.

Prediction of GI toxicity. A baseline pCLE examination performed before or at the initiation of ICI therapy could investigate whether pre-existing alterations in epithelial barrier-associated features are associated with the subsequent development of GI irAEs. This potential application is based on the hypothesis that differences in intestinal barrier integrity may contribute to individual susceptibility to ICI-related toxicity and may interact with the gut microbiota and mucosal immune environment. In this setting, pCLE could provide spatially resolved information on epithelial barrier-associated abnormalities that might complement microbiome profiling and circulating or fecal biomarkers.

It is important, however, to distinguish this potential predictive application from the evidence currently available. Our preliminary observations were obtained after the onset of clinically apparent GI toxicity and therefore provide no evidence that pCLE abnormalities precede symptoms or colitis. Accordingly, the hypothesis that pCLE abnormalities may identify patients at increased risk of subsequent GI toxicity remains to be tested and requires prospective longitudinal studies beginning before or at ICI initiation.

An additional methodological consideration is the timing of microbiome sampling relative to bowel preparation. Because bowel cleansing may alter the composition and detectability of intestinal microbial communities, prospective studies integrating microbiome profiling with pCLE should standardize stool collection timing and document the interval between sampling, bowel preparation, and endoscopic assessment. Recent antibiotic exposure should also be systematically considered when interpreting microbiome studies in ICI-treated patients. Antibiotics can induce substantial and sometimes prolonged alterations in microbial diversity and composition, potentially confounding associations between specific microbial signatures, intestinal barrier dysfunction, and ICI outcomes. Prospective studies should therefore record antibiotic type, indication, timing, and duration and incorporate these variables into statistical analyses.

The ICI regimen represents another important determinant of GI toxicity and should be considered when evaluating the clinical utility of pCLE. In particular, anti-CTLA-4-containing combinations are associated with a higher risk of GI irAEs than anti-PD-1/PD-L1 monotherapy. Future studies should therefore consider stratification by ICI regimen or incorporate treatment regimen as a covariate when evaluating the predictive performance of pCLE.

Diagnosis and characterization of GI toxicity. Once GI symptoms develop, pCLE could potentially complement conventional endoscopy and histopathology during the evaluation of suspected ICI-associated enterocolitis. Conventional endoscopy provides information on macroscopic mucosal changes, while histopathology characterizes tissue inflammation and structural alterations. pCLE may add spatially resolved in vivo information on epithelial and microvascular features associated with barrier dysfunction and inflammation, including fluorescein leakage, epithelial architectural alterations, crypt abnormalities, and vascular leakage. This complementary information may be particularly relevant in areas with limited or equivocal macroscopic abnormalities. Nevertheless, pCLE should not be considered a substitute for histopathological assessment, and its diagnostic performance in ICI-treated patients remains to be established.

An important aspect of this potential application is the interpretation of discordant findings between pCLE and conventional assessment. For example, pCLE-detected epithelial barrier-associated abnormalities in the absence of histological inflammation may reflect a functional alteration that is not captured by tissue sampling, but may also result from spatial heterogeneity or limitations in the interpretation of fluorescein leakage. Conversely, histological inflammation in an area without evident pCLE abnormalities may reflect differences in the distribution or nature of the inflammatory process, or limitations related to the restricted field of pCLE imaging. Prospective studies should therefore investigate pCLE and histopathology as complementary rather than interchangeable assessments.

Monitoring of mucosal recovery. Repeated pCLE examinations during follow-up could provide longitudinal information on the persistence, resolution, or evolution of epithelial and microvascular abnormalities after treatment of ICI-associated GI toxicity. This may complement clinical assessment, endoscopy, histopathology, and fecal inflammatory markers by providing information on barrier-associated changes at the microscopic level. The observation that pCLE abnormalities may persist in some patients despite apparent endoscopic and histological remission provides a rationale for investigating whether functional recovery of the epithelial barrier follows a different trajectory from conventional measures of mucosal inflammation. Whether persistent abnormalities are associated with symptom recurrence, prolonged inflammation, or response to corticosteroid tapering remains unknown.

### 5.3. Limitations and Future Perspectives for pCLE in ICI-Induced Colitis

Despite its considerable potential, several limitations currently restrict the widespread implementation of pCLE in clinical practice. First, pCLE is a highly operator-dependent technique that requires dedicated training for both image acquisition and interpretation. Although standardized classifications have been developed for several GI diseases, universally accepted criteria for evaluating epithelial barrier dysfunction, particularly in the context of ICI-induced colitis, are still lacking. This highlights the need for validated scoring systems and multicenter studies to improve reproducibility and interobserver agreement.

A further requirement for prospective validation will be the pre-specification of a pCLE threshold defining clinically relevant barrier dysfunction. Because no validated cutoff currently exists for ICI-treated patients, threshold selection should ideally be established in a dedicated training cohort and subsequently tested in an independent validation cohort. Importantly, thresholds may need to be site-specific and should be evaluated against clinically meaningful endpoints, such as the occurrence, severity, and persistence of GI irAEs.

A crucial point to consider is that the interval between intravenous fluorescein administration and image acquisition may influence the appearance and interpretation of fluorescein leakage and is therefore relevant for the standardization of pCLE assessment. In our protocol, pCLE imaging was performed within 10 min after intravenous fluorescein administration. Nevertheless, because the intensity and distribution of fluorescein signal may vary according to the time elapsed after intravenous administration, the interval between fluorescein injection and image acquisition should be standardized in prospective studies to improve reproducibility and facilitate comparison between examinations. Of note, fluorescein leakage should currently be considered an imaging surrogate of altered barrier integrity rather than a direct quantitative measurement of transepithelial permeability.

A second limitation is the restricted field of view. Unlike conventional endoscopy, pCLE evaluates only small areas of the mucosa, making the examination susceptible to sampling bias, particularly in diseases characterized by patchy or heterogeneous involvement. Spatial heterogeneity may also contribute to discordance between pCLE findings and histopathological assessment when the two techniques sample different microscopic areas. Furthermore, image acquisition increases procedural time.

From a practical perspective, the availability of pCLE remains limited to specialized referral centers because of the high cost of the equipment and the need for dedicated expertise. Consequently, the evidence supporting its clinical utility in ICI-treated patients remains limited, and data are currently derived mainly from small exploratory or single-center studies. In addition, although pCLE provides in vivo microscopic information on fluorescence patterns and morphological features associated with epithelial barrier integrity, epithelial architecture, and microvascular alterations, it should currently be regarded as complementary rather than alternative to conventional endoscopy and histopathology.

Finally, integration of pCLE into routine clinical practice will require prospective, adequately powered multicenter studies establishing the reproducibility and clinical meaning of pCLE findings and determining their diagnostic, predictive, and prognostic value. Future investigations should establish standardized acquisition and interpretation protocols, establish the reproducibility and clinical meaning of pCLE findings, including the performance of pre-specified thresholds across independent cohorts, and explore quantitative image analysis, including artificial intelligence-assisted approaches. Importantly, pCLE should also be investigated within an integrated framework combining microbiome profiling, nutritional assessment, circulating and fecal biomarkers, histopathology, and longitudinal clinical outcomes. Such studies could determine whether pCLE-detected barrier-associated abnormalities provide complementary information for risk stratification, diagnosis, or monitoring of ICI-associated gastrointestinal toxicity and whether their integration with microbiome and host-related variables can improve predictive models for precision immuno-oncology.

**Figure 3:**
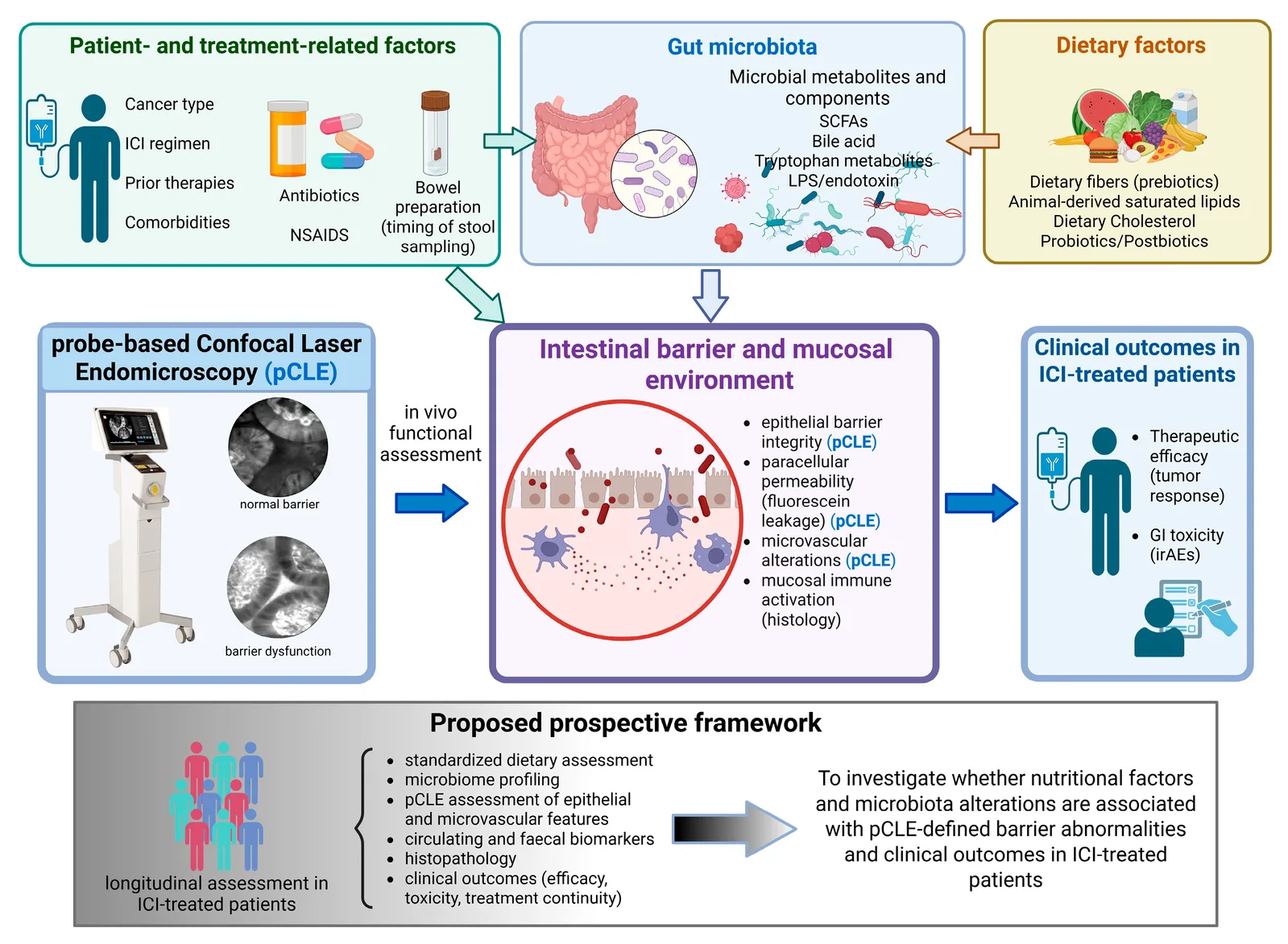
Proposed integrative framework linking gut microbiota, intestinal barrier integrity, pCLE, and clinical outcomes during immune checkpoint inhibitor (ICI) therapy. Patient- and treatment-related factors, including cancer characteristics, ICI regimen, concomitant medications, antibiotic exposure, and bowel preparation, together with dietary factors, may influence gut microbiota composition and function. Microbial metabolites and components may contribute to modulation of intestinal barrier integrity and mucosal immune responses. Probe-based confocal laser endomicroscopy (pCLE) represents the functional imaging component of the proposed framework, enabling real-time, in vivo assessment of epithelial barrier-associated abnormalities, vascular permeability, and mucosal microarchitecture. These imaging features may complement microbiome profiling, nutritional assessment, circulating and fecal biomarkers, and histopathology in relation to clinical outcomes, including ICI efficacy and GI irAEs. The lower panel illustrates a proposed prospective longitudinal framework integrating standardized clinical and treatment data, dietary assessment, microbiome profiling, pCLE, biomarkers, histopathology, and clinical outcomes. This framework is hypothesis-generating: direct evidence linking microbiota alterations, pCLE-detected barrier abnormalities, and ICI outcomes is currently lacking, and the proposed relationships should be evaluated prospectively without presupposing causality.

Based on the evidence and knowledge gaps discussed above, we propose an integrated prospective framework combining pCLE with nutritional assessment, microbiome profiling, biomarkers, histopathology, and longitudinal clinical outcomes (Figure 3).

## 6. Conclusions and Future Perspective

Gut microbiota composition and intestinal barrier integrity are increasingly recognized as potentially important components of the biological context influencing ICI efficacy and toxicity; however, their temporal and causal relationships with treatment outcomes remain incompletely defined. Although considerable progress has been made in identifying microbial signatures associated with clinical outcomes, the functional assessment of epithelial barrier integrity remains an underexplored area in cancer immunotherapy. In this context, pCLE represents a promising technology capable of providing real-time, in vivo visualization of epithelial permeability and mucosal architecture, thereby offering information that complements conventional endoscopy, histopathology, and microbiome profiling.

By enabling the detection of epithelial barrier-associated abnormalities that may not be apparent on conventional assessment, pCLE has the potential to improve patient stratification, potentially identify individuals at increased risk of immune-related GI toxicity, and provide novel insights into the mechanisms linking the gut microbiota, intestinal permeability, and antitumor immunity. Furthermore, longitudinal assessment of barrier integrity may help monitor mucosal healing and support clinical decisions regarding corticosteroid tapering, treatment optimization, and the safe resumption of immunotherapy.

Although prospective clinical validation is still required, integrating functional imaging with microbiome analysis, circulating biomarkers, and clinical parameters could establish a new multimodal approach for precision immuno-oncology. Such a strategy may enable more personalized therapeutic management, reducing unnecessary treatment discontinuation and avoiding ineffective interventions while optimizing healthcare resource utilization. Ultimately, pCLE has the potential to bridge the gap between basic insights into gut barrier biology and their translation into clinically actionable biomarkers, paving the way for a more individualized approach to cancer immunotherapy.

Future prospective multicenter studies integrating pCLE with microbiome profiling, host immune signatures, and nutritional interventions will be essential to determine whether preservation of intestinal barrier integrity can serve not only as a biomarker of treatment response and toxicity but also as a therapeutic target for optimizing cancer immunotherapy.

## Data Availability

The present article is a mini-review and does not report a formal analysis of a dedicated research dataset. Preliminary proof-of-concept clinical observations are mentioned for illustrative purposes, and the corresponding unpublished patient-derived images shown in Figure 1 and Figure 2 are not part of a publicly available dataset. Therefore, data sharing is not applicable to this article.

## Abbreviations

The following abbreviations are used in this manuscript:

ICI	Immune Checkpoint Inhibitor
pCLE	Probe-based Confocal Laser Endomicroscopy
IBD	Inflammatory Bowel Disease
GI	gastrointestinal
irAE	immune-related adverse event
SCFA	short-chain fatty acid
PD-1	programmed cell death protein 1
PD-L1	programmed death-ligand 1
CTLA-4	cytotoxic T-lymphocyte-associated protein 4

## References

[1] Palmeri M., Mehnert J., Silk A.W., Jabbour S.K., Ganesan S., Popli P., Riedlinger G., Stephenson R., de Meritens A.B., Leiser A. (2022). Real-World Application of Tumor Mutational Burden-High (TMB-High) and Microsatellite Instability (MSI) Confirms Their Utility as Immunotherapy Biomarkers. ESMO Open.

[2] Basile D., Garattini S.K., Bonotto M., Ongaro E., Casagrande M., Cattaneo M., Fanotto V., De Carlo E., Loupakis F., Urbano F. (2017). Immunotherapy for Colorectal Cancer: Where Are We Heading?. Expert Opin. Biol. Ther..

[3] Bonotto M., Garattini S.K., Basile D., Ongaro E., Fanotto V., Cattaneo M., Cortiula F., Iacono D., Cardellino G.G., Pella N. (2017). Immunotherapy for Gastric Cancers: Emerging Role and Future Perspectives. Expert Rev. Clin. Pharmacol..

[4] Chen P.-L., Roh W., Reuben A., Cooper Z.A., Spencer C.N., Prieto P.A., Miller J.P., Bassett R.L., Gopalakrishnan V., Wani K. (2016). Analysis of Immune Signatures in Longitudinal Tumor Samples Yields Insight into Biomarkers of Response and Mechanisms of Resistance to Immune Checkpoint Blockade. Cancer Discov..

[5] Esfahani K., Roudaia L., Buhlaiga N., Del Rincon S.V., Papneja N., Miller W.H. (2020). A Review of Cancer Immunotherapy: From the Past, to the Present, to the Future. Curr. Oncol..

[6] Postow M.A., Sidlow R., Hellmann M.D. (2018). Immune-Related Adverse Events Associated with Immune Checkpoint Blockade. N. Engl. J. Med..

[7] Dougan M., Wang Y., Rubio-Tapia A., Lim J.K. (2021). AGA Clinical Practice Update on Diagnosis and Management of Immune Checkpoint Inhibitor Colitis and Hepatitis: Expert Review. Gastroenterology.

[8] Johnson D.B., Nebhan C.A., Moslehi J.J., Balko J.M. (2022). Immune-Checkpoint Inhibitors: Long-Term Implications of Toxicity. Nat. Rev. Clin. Oncol..

[9] Wang D.Y., Salem J.-E., Cohen J.V., Chandra S., Menzer C., Ye F., Zhao S., Das S., Beckermann K.E., Ha L. (2018). Fatal Toxic Effects Associated with Immune Checkpoint Inhibitors: A Systematic Review and Meta-analysis. JAMA Oncol..

[10] Favara D.M., Spain L., Au L., Clark J., Daniels E., Diem S., Chauhan D., Turajlic S., Powell N., Larkin J.M. (2020). Five-Year Review of Corticosteroid Duration and Complications in the Management of Immune Checkpoint Inhibitor-Related Diarrhoea and Colitis in Advanced Melanoma. ESMO Open.

[11] Derosa L., Iebba V., Silva C.A.C., Piccinno G., Wu G., Lordello L., Routy B., Zhao N., Thelemaque C., Birebent R. (2024). Custom Scoring Based on Ecological Topology of Gut Microbiota Associated with Cancer Immunotherapy Outcome. Cell.

[12] Gopalakrishnan V., Spencer C.N., Nezi L., Reuben A., Andrews M.C., Karpinets T.V., Prieto P.A., Vicente D., Hoffman K., Wei S.C. (2018). Gut Microbiome Modulates Response to Anti-PD-1 Immunotherapy in Melanoma Patients. Science.

[13] Gunjur A., Shao Y., Rozday T., Klein O., Mu A., Haak B.W., Markman B., Kee D., Carlino M.S., Underhill C. (2024). A Gut Microbial Signature for Combination Immune Checkpoint Blockade across Cancer Types. Nat. Med..

[14] Hayase E., Jenq R.R. (2021). Role of the Intestinal Microbiome and Microbial-Derived Metabolites in Immune Checkpoint Blockade Immunotherapy of Cancer. Genome Med..

[15] Andrews M.C., Duong C.P.M., Gopalakrishnan V., Iebba V., Chen W.-S., Derosa L., Khan M.A.W., Cogdill A.P., White M.G., Wong M.C. (2021). Gut Microbiota Signatures Are Associated with Toxicity to Combined CTLA-4 and PD-1 Blockade. Nat. Med..

[16] Coutzac C., Jouniaux J.-M., Paci A., Schmidt J., Mallardo D., Seck A., Asvatourian V., Cassard L., Saulnier P., Lacroix L. (2020). Systemic Short Chain Fatty Acids Limit Antitumor Effect of CTLA-4 Blockade in Hosts with Cancer. Nat. Commun..

[17] Frankel A.E., Coughlin L.A., Kim J., Froehlich T.W., Xie Y., Frenkel E.P., Koh A.Y. (2017). Metagenomic Shotgun Sequencing and Unbiased Metabolomic Profiling Identify Specific Human Gut Microbiota and Metabolites Associated with Immune Checkpoint Therapy Efficacy in Melanoma Patients. Neoplasia.

[18] Li S., Che C., Zhou Y., Fan D., Bai X., Lu Y., Zhao X. (2026). Gut Microecology Empowers Cancer Immunotherapy: Commensal Microbiota-Mediated Mechanisms and Translational Prospects of PD-1/PD-L1 Therapy. Cancer Biol. Med..

[19] Liu K., Xue X., Qin H., Zhu J., Jin M., Dai D., Tang Y., Bukhari I., Liu H., Qiu C. (2026). Gut Associated Metabolites Enhance PD-L1 Blockade Efficacy in Prostate Cancer. Oncol. Res..

[20] Routy B., Le Chatelier E., Derosa L., Duong C.P.M., Alou M.T., Daillère R., Fluckiger A., Messaoudene M., Rauber C., Roberti M.P. (2018). Gut Microbiome Influences Efficacy of PD-1-Based Immunotherapy against Epithelial Tumors. Science.

[21] Mann E.R., Lam Y.K., Uhlig H.H. (2024). Short-Chain Fatty Acids: Linking Diet, the Microbiome and Immunity. Nat. Rev. Immunol..

[22] Parada Venegas D., De la Fuente M.K., Landskron G., González M.J., Quera R., Dijkstra G., Harmsen H.J.M., Faber K.N., Hermoso M.A. (2019). Short Chain Fatty Acids (SCFAs)-Mediated Gut Epithelial and Immune Regulation and Its Relevance for Inflammatory Bowel Diseases. Front. Immunol..

[23] Baruch E.N., Youngster I., Ben-Betzalel G., Ortenberg R., Lahat A., Katz L., Adler K., Dick-Necula D., Raskin S., Bloch N. (2021). Fecal Microbiota Transplant Promotes Response in Immunotherapy-Refractory Melanoma Patients. Science.

[24] Duttagupta S., Messaoudene M., Hunter S., Desilets A., Jamal R., Mihalcioiu C., Belkaid W., Marcoux N., Fidelle M., Suissa D. (2026). Fecal Microbiota Transplantation plus Immunotherapy in Non-Small Cell Lung Cancer and Melanoma: The Phase 2 FMT-LUMINate Trial. Nat. Med..

[25] Neurath M.F., Artis D., Becker C. (2025). The Intestinal Barrier: A Pivotal Role in Health, Inflammation, and Cancer. Lancet Gastroenterol. Hepatol..

[26] Chelakkot C., Choi Y., Kim D.-K., Park H.T., Ghim J., Kwon Y., Jeon J., Kim M.-S., Jee Y.-K., Gho Y.S. (2018). Akkermansia Muciniphila-Derived Extracellular Vesicles Influence Gut Permeability through the Regulation of Tight Junctions. Exp. Mol. Med..

[27] Peterson L.W., Artis D. (2014). Intestinal Epithelial Cells: Regulators of Barrier Function and Immune Homeostasis. Nat. Rev. Immunol..

[28] Iacucci M., Santacroce G., Majumder S., Morael J., Zammarchi I., Maeda Y., Ryan D., Di Sabatino A., Rescigno M., Aburto M.R. (2024). Opening the Doors of Precision Medicine: Novel Tools to Assess Intestinal Barrier in Inflammatory Bowel Disease and Colitis-Associated Neoplasia. Gut.

[29] Hone Lopez S., Jalving M., Fehrmann R.S.N., Nagengast W.B., de Vries E.G.E., de Haan J.J. (2022). The Gut Wall’s Potential as a Partner for Precision Oncology in Immune Checkpoint Treatment. Cancer Treat. Rev..

[30] Cannizzaro R., Mongiat M., Canzonieri V., Fornasarig M., Maiero S., De Re V., Todaro F., De Paoli P., Spessotto P. (2012). Endomicroscopy and Cancer: A New Approach to the Visualization of Neoangiogenesis. Gastroenterol. Res. Pract..

[31] Capuano A., Andreuzzi E., Pivetta E., Doliana R., Favero A., Canzonieri V., Maiero S., Fornasarig M., Magris R., Cannizzaro R. (2019). The Probe Based Confocal Laser Endomicroscopy (pCLE) in Locally Advanced Gastric Cancer: A Powerful Technique for Real-Time Analysis of Vasculature. Front. Oncol..

[32] Spessotto P., Clemente N., Mongiat M., Capuano A., Baldassarre G., Polesel J., Del Fabro A., Lucia E., Realdon S., Maiero S. (2025). Probe-Based Confocal Laser Endomicroscopy Intra-Operative Evaluation in Ovarian Cancer: Definition of in Vivo Architectural Patterns to Determine Resection Strategies. Int. J. Gynecol. Cancer.

[33] Spessotto P., Fornasarig M., Pivetta E., Maiero S., Magris R., Mongiat M., Canzonieri V., De Paoli P., De Paoli A., Buonadonna A. (2017). Probe-Based Confocal Laser Endomicroscopy for in Vivo Evaluation of the Tumor Vasculature in Gastric and Rectal Carcinomas. Sci. Rep..

[34] Kiesslich R., Duckworth C.A., Moussata D., Gloeckner A., Lim L.G., Goetz M., Pritchard D.M., Galle P.R., Neurath M.F., Watson A.J.M. (2012). Local Barrier Dysfunction Identified by Confocal Laser Endomicroscopy Predicts Relapse in Inflammatory Bowel Disease. Gut.

[35] Lenti M.V., Rossi C.M., Cannizzaro R., Santacroce G., Lizier M., Fornasa G., Merli S., Pasini A., Savarino E.V., Spessotto P. (2025). Disrupted Mucosal Vascular Barrier in Eosinophilic Esophagitis. Sci. Rep..

[36] Spessotto P., Piacentini A., Rebuzzi R., Rossi D., Zdjelar A., Cigana C., Crestani S., Michieli M., Savarino E.V., Di Sabatino A. (2026). Probing the Gut Barrier: Epithelial Permeability as a Dynamic Marker in Eosinophilic Gastroenteritis via pCLE. Ther. Adv. Gastrointest. Endosc..

[37] Horowitz A., Chanez-Paredes S.D., Haest X., Turner J.R. (2023). Paracellular Permeability and Tight Junction Regulation in Gut Health and Disease. Nat. Rev. Gastroenterol. Hepatol..

[38] van der Post S., Jabbar K.S., Birchenough G., Arike L., Akhtar N., Sjovall H., Johansson M.E.V., Hansson G.C. (2019). Structural Weakening of the Colonic Mucus Barrier Is an Early Event in Ulcerative Colitis Pathogenesis. Gut.

[39] Shan Y., Zheng M., Zhao Y., Sha S., Bian C. (2026). Synbiotics Regulate Gut Microbiota-Derived Indole-3-Acetic Acid Production to Modulate the Intestinal Barrier and Improve Antibiotic-Associated Diarrhea. J. Dairy Sci..

[40] Li D.K., Chaudhari S.N., Lee Y., Sojoodi M., Adhikari A.A., Zukerberg L., Shroff S., Barrett S.C., Tanabe K., Chung R.T. (2022). Inhibition of Microbial Deconjugation of Micellar Bile Acids Protects against Intestinal Permeability and Liver Injury. Sci. Adv..

[41] Park N., Lee B., Jeon H.-J., Yeon Kim J., Park S., Kim S.-E., Lee D.-K., Lee Y. (2026). Inulin-Butyrate Nanogel for Modulation of Gut Microbiome, Intestinal Barrier, and Regulatory T-Cells in Colitis. Small.

[42] Jin C., Gao X., Zhang F., Liu Y., Yu J., Mu G., Tuo Y. (2026). Anti-Inflammatory and Barrier-Protective Effects of Metabolites from Lactobacillus Co-Fermentation with Linoleic Acid and Human Fecal Microbiota. Food Res. Int..

[43] Sasson S.C., Slevin S.M., Cheung V.T.F., Nassiri I., Olsson-Brown A., Fryer E., Ferreira R.C., Trzupek D., Gupta T., Al-Hillawi L. (2021). Interferon-Gamma-Producing CD8^+^ Tissue Resident Memory T Cells Are a Targetable Hallmark of Immune Checkpoint Inhibitor-Colitis. Gastroenterology.

[44] Thomas M.F., Slowikowski K., Manakongtreecheep K., Sen P., Samanta N., Tantivit J., Nasrallah M., Zubiri L., Smith N.P., Tirard A. (2024). Single-Cell Transcriptomic Analyses Reveal Distinct Immune Cell Contributions to Epithelial Barrier Dysfunction in Checkpoint Inhibitor Colitis. Nat. Med..

[45] Reschke R., Shapiro J.W., Yu J., Rouhani S.J., Olson D.J., Zha Y., Gajewski T.F. (2022). Checkpoint Blockade-Induced Dermatitis and Colitis Are Dominated by Tissue-Resident Memory T Cells and Th1/Tc1 Cytokines. Cancer Immunol. Res..

[46] He Y., Fu L., Li Y., Wang W., Gong M., Zhang J., Dong X., Huang J., Wang Q., Mackay C.R. (2021). Gut Microbial Metabolites Facilitate Anticancer Therapy Efficacy by Modulating Cytotoxic CD8^+^ T Cell Immunity. Cell Metab..

[47] Malik A., Sharma D., Aguirre-Gamboa R., McGrath S., Zabala S., Weber C., Jabri B. (2023). Epithelial IFNγ Signalling and Compartmentalized Antigen Presentation Orchestrate Gut Immunity. Nature.

[48] Zeng L., Wang Y., Shen J., Wei X., Wu Y., Chi X., Zheng X., Yu X., Shi Y., Liu W. (2024). TIPE2 Aggravates Experimental Colitis and Disrupts Intestinal Epithelial Barrier Integrity by Activating JAK2/STAT3/SOCS3 Signal Pathway. Exp. Cell Res..

[49] Drymel B., Tomela K., Galus Ł., Olejnik-Schmidt A., Mackiewicz J., Kaczmarek M., Mackiewicz A., Schmidt M. (2025). Characteristics of Intestinal Barrier State and Immunoglobulin-Bound Fraction of Stool Microbiota in Advanced Melanoma Patients Undergoing Anti-PD-1 Therapy. Int. J. Mol. Sci..

[50] Xiong L., Huang J., Dong Y., Han W., Kuo W.-T., Xu W., Han Y., An C., Zhu R., Zhu N. (2026). Targeting MLCK1 Uncouples Immune Checkpoint Inhibitor-Induced Colitis from Antitumour Immunity. Gut.

[51] Cheung V.T.F., Gupta T., Olsson-Brown A., Subramanian S., Sasson S.C., Heseltine J., Fryer E., Collantes E., Sacco J.J., Pirmohamed M. (2020). Immune Checkpoint Inhibitor-Related Colitis Assessment and Prognosis: Can IBD Scoring Point the Way?. Br. J. Cancer.

[52] Zhang Y., Xu X., Wang S., Yin X., Zhang B., Zhu Z., Ji R., Zhu J., He H., Cheng S. (2026). Fecal Microbiota Transplantation Combined with Anti-PD-1 Therapy in Refractory Microsatellite-Stable Gastric Cancer: A Phase I Feasibility and Safety Study. J. Immunother. Cancer.

[53] Zhang E., Kiely C., Sandanayake N., Tattersall S. (2021). Calcineurin Inhibitors in Steroid and Anti-TNF-Alpha Refractory Immune Checkpoint Inhibitor Colitis. JGH Open.

[54] Ishihara H., Watanabe T., Kumei S., Kume K., Yoshikawa I., Harada M. (2024). A Case of Refractory Immune Checkpoint Inhibitor-Induced Colitis Improved by the Treatment with Vedolizumab and Granulocyte-Monocyte Apheresis Combination Therapy. Clin. J. Gastroenterol..

[55] Perez-Ruiz E., Minute L., Otano I., Alvarez M., Ochoa M.C., Belsue V., de Andrea C., Rodriguez-Ruiz M.E., Perez-Gracia J.L., Marquez-Rodas I. (2019). Prophylactic TNF Blockade Uncouples Efficacy and Toxicity in Dual CTLA-4 and PD-1 Immunotherapy. Nature.

[56] Wang Y., Wiesnoski D.H., Helmink B.A., Gopalakrishnan V., Choi K., DuPont H.L., Jiang Z.-D., Abu-Sbeih H., Sanchez C.A., Chang C.-C. (2018). Fecal Microbiota Transplantation for Refractory Immune Checkpoint Inhibitor-Associated Colitis. Nat. Med..

[57] Abu-Sbeih H., Ali F.S., Alsaadi D., Jennings J., Luo W., Gong Z., Richards D.M., Charabaty A., Wang Y. (2018). Outcomes of Vedolizumab Therapy in Patients with Immune Checkpoint Inhibitor-Induced Colitis: A Multi-Center Study. J. Immunother. Cancer.

[58] Huang C., Li M., Liu B., Zhu H., Dai Q., Fan X., Mehta K., Huang C., Neupane P., Wang F. (2021). Relating Gut Microbiome and Its Modulating Factors to Immunotherapy in Solid Tumors: A Systematic Review. Front. Oncol..

[59] Fernandes R., Jabbarizadeh B., Rajeh A., Hong M.M.Y., Baines K.J., Ernst S., Winquist E., Ali A.S., Penny S., Figueredo R. (2026). Fecal Microbiota Transplantation plus Immunotherapy in Metastatic Renal Cell Carcinoma: The Phase 1 PERFORM Trial. Nat. Med..

[60] Chen J., Li W., Yang L., Li J., Wang S., Chen Z., Xu S., Wen M., Liang J., Hu Z. (2025). Microbiota-Derived Butyrate Potentiates MSLN CAR-T Cell Therapy by Metabolic Reprogramming and Extracellular Matrix Remodeling. Biomed. Pharmacother..

[61] Lin A., Huang L., Jiang A., Zhu L., Mou W., Li Y., Zhang C., Liu Z., Zhang J., Cheng Q. (2025). Microbiota Boost Immunotherapy? A Meta-Analysis Dives into Fecal Microbiota Transplantation and Immune Checkpoint Inhibitors. BMC Med..

[62] Bolte L.A., Björk J.R., Gacesa R., Weersma R.K. (2025). Pharmacomicrobiomics: The Role of the Gut Microbiome in Immunomodulation and Cancer Therapy. Gastroenterology.

[63] Matson V., Fessler J., Bao R., Chongsuwat T., Zha Y., Alegre M.-L., Luke J.J., Gajewski T.F. (2018). The Commensal Microbiome Is Associated with Anti-PD-1 Efficacy in Metastatic Melanoma Patients. Science.

[64] Fukuhara M., Sahay B., Fanger G.R., Freguia C.F. (2026). R-5780, a SagA-Engineered *Lactococcus lactis*, Is a Safe Oral Synthetic-Biology Microbial Therapy That Potentiates PD-1 Blockade. Cancer Immunol. Immunother..

[65] Lee J.B., Baek S., Kim D.K., Kwon B.-E., Ahn J.S., Nagasaka M., Davar D., Park H., Kim H., Im J. (2026). Phase I Trial of CJRB-101 plus Pembrolizumab in Patients with Metastatic Non-Small Cell Lung Cancer, Head and Neck Squamous Cell Carcinoma and Melanoma. J. Immunother. Cancer.

[66] Furusawa Y., Obata Y., Fukuda S., Endo T.A., Nakato G., Takahashi D., Nakanishi Y., Uetake C., Kato K., Kato T. (2013). Commensal Microbe-Derived Butyrate Induces the Differentiation of Colonic Regulatory T Cells. Nature.

[67] Rankin L.C., Kaiser K.A., de Los Santos-Alexis K., Park H., Uhlemann A.-C., Gray D.H.D., Arpaia N. (2023). Dietary Tryptophan Deficiency Promotes Gut RORγt^+^ Treg Cells at the Expense of Gata3^+^ Treg Cells and Alters Commensal Microbiota Metabolism. Cell Rep..

[68] Arpaia N., Campbell C., Fan X., Dikiy S., van der Veeken J., deRoos P., Liu H., Cross J.R., Pfeffer K., Coffer P.J. (2013). Metabolites Produced by Commensal Bacteria Promote Peripheral Regulatory T-Cell Generation. Nature.

[69] Malczewski A.B., Ketheesan N., Coward J.I.G., Navarro S. (2021). Enhancing Checkpoint Inhibitor Therapy in Solid Tissue Cancers: The Role of Diet, the Microbiome & Microbiome-Derived Metabolites. Front. Immunol..

[70] Zhou Q., Verne M.L., Fields J.Z., Lefante J.J., Basra S., Salameh H., Verne G.N. (2019). Randomised Placebo-Controlled Trial of Dietary Glutamine Supplements for Postinfectious Irritable Bowel Syndrome. Gut.

[71] Spencer C.N., McQuade J.L., Gopalakrishnan V., McCulloch J.A., Vetizou M., Cogdill A.P., Khan M.A.W., Zhang X., White M.G., Peterson C.B. (2021). Dietary Fiber and Probiotics Influence the Gut Microbiome and Melanoma Immunotherapy Response. Science.

[72] Mattavelli E., Da Prat V., Corallo S., Tartara A., Figini S., De Simeis F., Marino S., Uggè A., Caccialanza R., Pedrazzoli P. (2026). Harnessing the Gut Microbiota in Extra-Intestinal Cancers: From Causal Evidence to Immunotherapy Strategies. Immunotherapy.

[73] Turpin W., Lee S.-H., Raygoza Garay J.A., Madsen K.L., Meddings J.B., Bedrani L., Power N., Espin-Garcia O., Xu W., Smith M.I. (2020). Increased Intestinal Permeability Is Associated with Later Development of Crohn’s Disease. Gastroenterology.

[74] Camilleri M. (2019). Leaky Gut: Mechanisms, Measurement and Clinical Implications in Humans. Gut.

[75] Seethaler B., Basrai M., Neyrinck A.M., Nazare J.-A., Walter J., Delzenne N.M., Bischoff S.C. (2021). Biomarkers for Assessment of Intestinal Permeability in Clinical Practice. Am. J. Physiol. Gastrointest. Liver Physiol..

[76] Adriaanse M.P.M., Tack G.J., Passos V.L., Damoiseaux J.G.M.C., Schreurs M.W.J., van Wijck K., Riedl R.G., Masclee A.A.M., Buurman W.A., Mulder C.J.J. (2013). Serum I-FABP as Marker for Enterocyte Damage in Coeliac Disease and Its Relation to Villous Atrophy and Circulating Autoantibodies. Aliment. Pharmacol. Ther..

[77] Hueso T., Gauthier J., Joncquel Chevalier-Curt M., Magro L., Coiteux V., Dulery R., Carpentier B., Labreuche J., Damaj G., Yakoub-Agha I. (2018). Association Between Low Plasma Level of Citrulline Before Allogeneic Hematopoietic Cell Transplantation and Severe Gastrointestinal Graft vs Host Disease. Clin. Gastroenterol. Hepatol..

[78] Schellekens D.H., Hundscheid I.H., Leenarts C.A., Grootjans J., Lenaerts K., Buurman W.A., Dejong C.H., Derikx J.P. (2017). Human Small Intestine Is Capable of Restoring Barrier Function after Short Ischemic Periods. World J. Gastroenterol..

[79] Nojkov B., Zhou S.-Y., Dolan R.D., Davis E.M., Appelman H.D., Guo X., Jackson K., Sturm M.B., Wang T.D., Owyang C. (2020). Evidence of Duodenal Epithelial Barrier Impairment and Increased Pyroptosis in Patients with Functional Dyspepsia on Confocal Laser Endomicroscopy and “Ex Vivo” Mucosa Analysis. Am. J. Gastroenterol..

[80] Barbaro M.R., Cremon C., Marasco G., Savarino E., Guglielmetti S., Bonomini F., Palombo M., Fuschi D., Rotondo L., Mantegazza G. (2024). Molecular Mechanisms Underlying Loss of Vascular and Epithelial Integrity in Irritable Bowel Syndrome. Gastroenterology.

[81] Gomez V., Buchner A.M., Dekker E., van den Broek F.J., Meining A., Shahid M.W., Ghabril M.S., Fockens P., Heckman M.G., Wallace M.B. (2010). Interobserver Agreement and Accuracy among International Experts with Probe-Based Confocal Laser Endomicroscopy in Predicting Colorectal Neoplasia. Endoscopy.

[82] Kuiper T., van den Broek F.J., van E.S., Wallace M.B., Buchner A.M., Meining A., van H.K., Fockens P., Dekker E. (2011). New Classification for Probe-Based Confocal Laser Endomicroscopy in the Colon. Endoscopy.

[83] Mascolo M., Staibano S., Ilardi G., Siano M., Vecchione M.L., Esposito D., De R.G., De Palma G.D. (2012). Probe-Based Confocal Laser Endomicroscopy Evaluation of Colon Preneoplastic Lesions, with Particular Attention to the Aberrant Crypt Foci, and Comparative Assessment with Histological Features Obtained by Conventional Endoscopy. Gastroenterol. Res. Pract..

[84] Park C.H., Kim H., Jo J.H., Hahn K.Y., Yoon J.-H., Kim S.Y., Lee Y.C., Noh S.H., Chung H.C., Lee S.K. (2019). Role of Probe-Based Confocal Laser Endomicroscopy-Targeted Biopsy in the Molecular and Histopathological Study of Gastric Cancer. J. Gastroenterol. Hepatol..

[85] Wang K.K., Carr-Locke D.L., Singh S.K., Neumann H., Bertani H., Galmiche J.P., Arsenescu R.I., Caillol F., Chang K.J., Chaussade S. (2015). Use of Probe-Based Confocal Laser Endomicroscopy (pCLE) in Gastrointestinal Applications. A Consensus Report Based on Clinical Evidence. United Eur. Gastroenterol. J..

[86] Zhang Y.L., Bai L., Li Z., Ji R., Li C.Q., Zuo X.L., Yin Y.F., Du J.X., Zhai Z.Z., Gao X.Z. (2016). Lower Dose of Fluorescein Sodium Is More Suitable for Confocal Laser Endomicroscopy: A Feasibility Study. Gastrointest. Endosc..

[87] Fornasarig M., Capuano A., Maiero S., Pivetta E., Guarnieri G., Canzonieri V., Zucchetto A., Mongiat M., Cannizzaro R., Spessotto P. (2021). pCLE Highlights Distinctive Vascular Patterns in Early Gastric Cancer and in Gastric Diseases with High Risk of Malignant Complications. Sci. Rep..

[88] Ferla S., Fucina S., Clemente N., Spessotto P., Cannizzaro R., Ditto A. (2026). Probe-Based Confocal Laser Endomicroscopy in Gynecologic Surgical Oncology: Intraoperative Insights to Guide Surgical Resection. Ann. Surg. Oncol..

[89] Iacucci M., Jeffery L., Acharjee A., Grisan E., Buda A., Nardone O.M., Smith S.C.L., Labarile N., Zardo D., Ungar B. (2023). Computer-Aided Imaging Analysis of Probe-Based Confocal Laser Endomicroscopy with Molecular Labeling and Gene Expression Identifies Markers of Response to Biological Therapy in IBD Patients: The Endo-Omics Study. Inflamm. Bowel Dis..

[90] Vieujean S., Atreya R., Buda A., Caradec J., Citi S., Danese S., Dewit O., Friedrich M., Ghosh S., Iacucci M. (2026). Tools and Advanced Imaging Technologies for Assessing Intestinal Epithelial Barrier Integrity: A Systematic Review. Am. J. Physiol. Gastrointest. Liver Physiol..

[91] Rath T., Atreya R., Bodenschatz J., Uter W., Geppert C.E., Vitali F., Fischer S., Waldner M.J., Colombel J.-F., Hartmann A. (2023). Intestinal Barrier Healing Is Superior to Endoscopic and Histologic Remission for Predicting Major Adverse Outcomes in Inflammatory Bowel Disease: The Prospective ERIca Trial. Gastroenterology.

